# Association of Control Service Types with Aviation Medical Examination Results and Sick Leave in Air Traffic Controllers in Korea

**DOI:** 10.3390/healthcare13020132

**Published:** 2025-01-12

**Authors:** Ju-Hyun Choi, Hye-Sun Jung, Eun-Hi Choi

**Affiliations:** 1 Department of Public Health, Graduate School, The Catholic University of Korea, Seoul 06591, Republic of Korea; juhyunchoi@catholic.ac.kr; 2 Department of Preventive Medicine, College of Medicine, The Catholic University of Korea, Seoul 06591, Republic of Korea; 3 Department of Nursing, College of Nursing, The Eulji University of Korea, Uijeongbu 13135, Republic of Korea

**Keywords:** air traffic controllers, aviation medical examination results, sick leave

## Abstract

**Objectives:** This study aimed to examine the association between the characteristics of air traffic controllers, their aviation medical examination results, and their sick leave, with the ultimate aim of promoting their health and contributing to the enhancement of aviation safety. **Methods**: The subjects of this study were air traffic controllers affiliated with the Ministry of Land, Infrastructure, and Transport and the Airport Corporation in various regions of Korea. Data collection was conducted through a survey from 10 May 2023 to 10 December 2023. **Results**: A total of 220 participants were included in the final analysis. An analysis of the factors associated with the aviation medical examination results revealed that approach controllers were 3.044 times more likely to receive a non-fit result (conditionally fit or unfit) compared to area controllers. An analysis of the factors associated with sick leave showed that approach controllers were 3.891 times more likely to take sick leave than area controllers. **Conclusions**: Based on the results of this study, it is necessary to implement regular job rotation to eliminate monotony, enhance job satisfaction, and provide diverse career experiences, considering the varying workloads and characteristics of different control tasks. Furthermore, various organizational support initiatives must be introduced to enhance both the physical and mental health of air traffic controllers.

## 1. Introduction

Global demand for air travel is steadily increasing, driven by rising income levels and the growth of low-cost carriers (LCCs). According to data from the Ministry of Land, Infrastructure, and Transport in Korea [1], although aviation demand temporarily stagnated due to the COVID-19 pandemic, air traffic in the fourth quarter of 2023 reached approximately 27.82 million passengers, with an average of over 2000 flights per day, indicating a continued upward trend. The number of professionals employed in the aviation industry is also steadily increasing. According to the 2021 Aviation Industry Survey in Korea, there were 7848 pilots and 1460 air traffic controllers (ATCs) [2].

ATCs are responsible for directing the takeoff and landing of aircraft carrying hundreds of passengers and monitoring flights for extended periods [3]. When they experience physical or mental health issues, they are unable to perform their duties [4]. This situation not only poses a personal concern but also presents a critical risk to public safety. Therefore, it is essential to effectively prevent and manage such health issues.

According to the Aviation Safety Act in Korea, aviation personnel, including pilots and air traffic controllers, are required to undergo periodic aviation medical examinations [5]. The results of these examinations, conducted at institutions registered as aviation medical examination facilities, are assessed by an aviation medical examiner who determines whether the individual is fit, conditionally fit, or unfit [6]. Additionally, those who have received certification from the aviation medical examination must report any deterioration in their physical or mental health to the Minister of Land, Infrastructure, and Transport in Korea, and they are prohibited from performing aviation duties until they are declared fit according to the medical standards [4]. Consequently, airlines and affiliated organizations implement appropriate sick leave policies, making the results of aviation medical examinations and the occurrence of sick leave critical factors in assessing the health status of air traffic controllers.

Due to the nature of air traffic control, which operates 24 h a day, long working hours and night shifts are known to induce fatigue and stress, thereby affecting job performance [7]. Studies have shown that when air traffic controllers experience significant physical and mental fatigue, their concentration decreases, leading to an increased risk of human error [8].

A survey conducted among shift-working air traffic controllers revealed that the most common reason for the difficulties associated with shift work was the irregularity of daily life, followed by lack of sleep, early morning shifts, and long working hours [9,10]. Additionally, it was noted that rotating shifts, which change daily, present a challenging working schedule, even for experienced controllers [10].

Evidence also suggests that, in addition to long working hours and night shifts, the air traffic service types affect fatigue differently. Approach control, which handles aircraft arrivals and departures, impacts fatigue due to its focus on conflict resolution and diverse job demands [11]. In contrast, area control involves more routine monitoring and reporting tasks [11].

Such physical changes are negatively associated with the results of aviation medical examinations. In cases where an ATC is deemed unfit, maintaining a valid certificate becomes impossible, posing challenges to career continuity [4]. For those deemed conditionally fit, regular treatment and consistent health management are required to maintain certification [12]. However, due to the nature of air traffic control service, managing health through individual efforts alone is challenging. The health management of air traffic controllers is essential not only for career maintenance and enhancing work efficiency but also for mitigating risks that could compromise aviation safety, necessitating both personal and organizational management [13].

A study on sick leave among flight crew members showed that, in addition to serious conditions that fail to meet aviation medical standards, minor illnesses such as colds, gastroenteritis, and otitis media are also common [14]. An analysis of sick leave duration revealed that most instances involved short-term leave of seven days or less [14]. Factors associated with sick leave include age, gender, and socioeconomic status [15]. Additionally, poor lifestyle habits and health conditions have been linked to increased sick leave and reduced productivity, leading to efforts to improve workers’ health through lifestyle modifications [12]. Regular health management and disease prevention are not only essential for preventing sick leave but also for enhancing productivity and ultimately ensuring aviation safety.

Alongside pilots, ATCs are essential personnel in the aviation industry. While research on human factors and aviation safety concerning ATCs is being conducted, studies on the factors affecting their health are not actively pursued both domestically and internationally. Therefore, this study aims to explore the association of control service types with aviation medical examination results and sick leave in ATCs.

## 2. Materials and Methods

### 2.1. Study Design

This is a descriptive cross-sectional study that aimed to identify factors associated with the results of aviation medical examinations (fit; non-fit) and the prevalence of sick leave of air traffic controllers (ATCs).

### 2.2. Participants

This is a secondary analysis study using data from the research project titled “Study on establishing a management system for the decline physical and mental condition of aviation workers” [13], commissioned by the Ministry of Land, Infrastructure, and Transport in Korea. After the Ministry of Land, Infrastructure, and Transport notified controllers nationwide about the research through an internal notice and obtained their consent, each individual accessed the online survey through the link they received and answered it. Data collection for this research was conducted from 10 May 2023 to 10 December 2023.

A total of 236 individuals participated in the survey. After excluding nine participants with missing data in the aviation medical examination results and seven participants who held an office position (flight information service) rather than field control service, 220 participants were included in this analysis.

### 2.3. Measurements

#### 2.3.1. Demographic Characteristics

The demographic characteristics comprised gender (male; female) and age (20s, 30s, and 40 and above).

#### 2.3.2. Occupational Characteristics

The occupational characteristics included working region, control service types, control service experience, working hours (weekly average), working schedule, and the number of night shifts (monthly average). Control service types were categorized into aerodrome control, approach control, area control, and flight information service. Aerodrome control pertains to tasks involving aircraft within the movement areas of an aerodrome and those flying in its vicinity. Approach control encompasses tasks related to aircraft during takeoff or landing phases, while area control focuses on maintaining separation between aircraft in flight to prevent mid-air collisions. Flight information service is responsible for providing the information and services necessary to ensure the safe and efficient navigation of aircraft [16]. ATCs for flight information service were excluded in this analysis. The working schedule excluded non-responses and consolidated the infrequent responses for the categories of 3 shifts and 4 shifts into a single classification. Consequently, the working schedule was categorized as 2 shifts, 3–4 shifts, and fixed day shift. The number of night shifts was classified into two categories: five or fewer days per month and six or more days per month.

#### 2.3.3. Health-Related Characteristics

The health-related characteristics consisted of hospital visit, reason for hospital visit, presence of health management, and health management types. Hospital visit indicated whether an individual had been to hospital to health issues in the past year. The reasons for hospital visit were categorized into treatment of existing diseases, evaluation of abnormal findings in aviation medical examinations, generalized fatigue, and abnormal physical symptoms. Due to the small sample size for the evaluation of abnormal findings in aviation medical examinations and generalized fatigue, these were categorized into a single classification. The presence of health management was assessed using 11 items deemed necessary for effective health management [13]. These items included smoking cessation, moderation in alcohol consumption, physical exercise, dietary improvement, stress management, weight control, management of musculoskeletal diseases, psychological counseling (mental health management), sleep management, fatigue management, and other relevant practices (e.g., medication). If an individual reported engaging in at least one of the eleven health management activities, they were classified as “yes”; otherwise, they were classified as “no”.

#### 2.3.4. Aviation Medical Examinations Results

Aviation medical examination results were categorized as either fit or non-fit. “Fit” referred to individuals deemed suitable following the health examination required for aviation personnel under the Aviation Safety Act in Korea, while “non-fit” included those classified as conditionally fit or unfit, excluding the fit category [6].

#### 2.3.5. Sick Leave

Sick leave was assessed in terms of its presence, duration, and reasons. The duration and reasons for sick leave were examined among those who had taken leave. The presence of sick leave indicated whether an individual had been unable to perform aviation duties due to health issues in the past year. The duration of sick leave referred to the number of days on leave, while the reasons for sick leave were categorized into respiratory diseases, gastrointestinal diseases, orthopedic diseases, otolaryngological diseases, and others (e.g., herpes zoster, thyroid disease, insomnia, etc.).

### 2.4. Data Analysis

The collected data were processed through error checking and coding, and statistical analyses were conducted using SPSS 25.0 software. A two-tailed significance level of *p* < 0.05 was employed as the criterion for statistical significance. Frequencies, percentages, means, and standard deviations were calculated to describe the characteristics of the participants. Differences in aviation medical examination results and the presence of sick leave based on the participants’ characteristics were analyzed using chi-square tests. Fisher’s exact test was employed instead of the chi-square test for variables with an expected count of less than 5. Binary logistic regression analysis was performed to assess the association of the participants’ characteristics with the aviation medical examination results (fit vs. non-fit) and the presence of sick leave.

### 2.5. Ethical Considerations

ATCs affiliated with the Ministry of Land, Infrastructure, and Transport and the Airport Corporation were informed of the study’s purpose and methods through a research information letter, and data were collected from those who voluntarily participated via an online survey. This study was conducted following final approval from the Institutional Review Board of E University in Korea (EUIRB2024-049).

## 3. Results

### 3.1. Demographic, Occupational, and Health-Related Characteristics

Among the participants, 53.6% were female, and 43.2% were aged 40 and above. Regarding working region, 61.8% were employed in Incheon. The distribution of control service types was as follows: 38.6% worked in area control, 33.2% in aerodrome control, and 28.2% in approach control. A plurality, 42.7%, had over 10 years of air traffic control service experience, and 52.7% reported an average weekly working time of 41 to 51 h. Regarding working schedules, 58.2% were engaged in two shifts, and a substantial majority, 84.5%, reported working more than six nights per month on average (Table 1).

There was a slightly higher proportion of individuals who did not visit the hospital (51.8%), with the primary reason for hospital visits being the treatment of existing diseases. In terms of health management, the majority of respondents reported actively managing their health, utilizing methods such as physical exercise (20.6%), smoking cessation (14.0%), and moderation in alcohol consumption (13.8%) (Table 1).

### 3.2. Characteristics of Aviation Medical Examination Results and Sick Leave

The most recent aviation medical examination results revealed that 81.4% of participants were classified as fit. Concerning sick leave, 83.6% reported no sick leave due to health problems in the past year, while those who had taken sick leave reported an average duration of 11.7 days (Mean ± SD 11.7 ± 13.7). Respiratory diseases accounted for 52.7% of the reasons for sick leave (Table 2).

### 3.3. Difference of Aviation Medical Examination Results by Demographic, Occupational, and Health-Related Characteristics

Among the occupational characteristics, control service types exhibited a significant difference in aviation medical examination results (χ^2^ = 13.472, *p* = 0.001). Participants engaged in aerodrome control were more likely to be classified as fit (Table 3).

The presence of health management utilized Fisher’s exact test rather than chi-square test to assess *p*-value due to the fact that 25% of all cells in the cross-analysis results had an expected count of less than 5. Consequently, there was no association between the presence of health management and the aviation medical examination results (Table 3).

### 3.4. Differences in the Presence of Sick Leave by Demographic, Occupational, and Health-Related Characteristics

Female participants exhibited a higher prevalence of sick leave (χ^2^ = 4.325, *p* = 0.038). Among the occupational characteristics, working region showed a difference prevalence in sick leave (χ^2^ = 10.410, *p* = 0.015). Control service types showed a significant difference in sick leave (χ^2^ = 7.988, *p* = 0.018). Approach controllers reported a greater frequency of sick leave (Table 4).

There was a statistically significant difference in the prevalence of sick leave when there was a history of hospital visit (χ^2^ = 24.802, *p* = 0.000). The presence of health management utilized Fisher’s exact test rather than chi-square test to assess *p*-value due to the fact that 25% of all cells in the cross-analysis results had an expected count of less than 5. Concerning health management types by multiple answers, certain variables demonstrated a difference prevalence in sick leave, including smoking cessation (χ^2^ = 4.086, *p* = 0.043), weight control (χ^2^ = 6.218, *p* = 0.013) and other relevant practices (χ^2^ = 10.316, *p* = 0.001) (Table 4).

### 3.5. Factors Associated with the Aviation Medical Examination Results (Fit vs. Non-Fit) of the Population

To explore the factors associated with the aviation medical examination results, logistic regression analysis was conducted using gender, control service task, control service experience, working hours, working schedule, and presence of health management as independent variables.

There was a significant correlation between age and control service experience, which was confirmed through the Cramer’s V coefficient indicating high multicollinearity in the correlation analysis. Additionally, a correlation was also observed between hospital visits and sick leave. Consequently, age and hospital visits were excluded from the input variables. Due to significant variations in the number of ATCs based on the size of each airport, the working region was excluded from this analysis, as it could introduce inaccuracies in the results. The model employed in this study was deemed appropriate based on the Hosmer–Lemeshow test.

The results of the logistic regression analysis indicated that the control service types were significantly associated with the aviation medical examination results. Specifically, approach controllers were 3.044 times (OR 3.044, 95% confidence interval [CI]: 1.278–7.250) more likely to receive a non-fit result (conditionally fit or unfit) compared to area controllers. Regarding working hours, ATCs with less than 40 h per week were 5.498 times (OR 5.498, 95% confidence interval [CI]: 1.451–20.840) more likely to receive a non-fit result (conditionally fit or unfit) compared to ATCs with over 52 h per week (Table 5).

### 3.6. Factors Associated with the Presence of Sick Leave Among the Population

To explore the factors associated with the presence of sick leave, logistic regression analysis was conducted using gender, control service task, control service experience, working hours, working schedule, and presence of health management as independent variables.

Age and hospital visit were excluded from the input variables after confirming high multicollinearity through Cramer’s V coefficient in the correlation analysis. Due to significant variations in the number of ATCs based on the size of each airport, the working region was excluded from this analysis, as it could introduce inaccuracies in the results. The model employed in this study was deemed appropriate based on the Hosmer–Lemeshow test.

The results of the logistic regression analysis revealed that control service types were significantly associated with the presence of sick leave. Specifically, approach controllers were 3.891 times (OR 3.891, 95% confidence interval [CI]: 1.461–10.364) more likely to take sick leave compared to area controllers. Otherwise, the working schedule was associated with the presence of sick leave, with two shifts showing a prevalence ratio of less than 1 when compared to having a fixed day shift (OR 0.155, 95% confidence interval [CI]: 0.025–0.973) (Table 6).

## 4. Discussion

This study aimed to investigate the association of ATCs’ characteristics with aviation medical examination results and the presence of sick leave.

It was found that the control service types were significantly associated with both the aviation medical examination results and the presence of sick leave in logistic regression analysis. For approach control, the likelihood of receiving a non-fit result (conditionally fit or unfit) was 3.044 times (OR 3.044, 95% confidence interval [CI]: 1.278–7.250) higher, and the prevalence of sick leave was 3.891 times (OR 3.891, 95% confidence interval [CI]: 1.461–10.364) greater compared to area control. The air traffic control process from takeoff to landing is divided into several stages: preflight, takeoff, departure, enroute, descent, approach, and landing. Among these, aviation accidents are known to occur most frequently during the takeoff, landing, and approach phases [17]. Airbus accident statistics from 2001 to 2020 reveal that a significant number of accidents occur during landing [18]. Approach controllers are tasked with ensuring the safe operation of aircraft during these high-risk phases, which inherently involves considerable pressure and responsibility [8]. Research has indicated that approach control, which focuses on conflict resolution during aircraft arrivals and departures, affects fatigue differently compared to area control, which involves routine monitoring and reporting tasks [11]. However, another study found that less experienced controllers and female workers tended to report lower perceived workload compared to other groups, although no significant difference in perceived workload was observed between area and approach controllers. It was concluded that air traffic controllers face high mental work demands, with certain individual factors influencing this workload [19].

A study on vessel traffic service operators (VTSOs), a profession similar to that of ATCs, revealed that VTSOs experienced higher levels of psychosocial stress compared to the general population, with stress levels following an inverted U-shape pattern based on age and work experience. Additionally, stress prevention education, improvements in the work environment, and the provision of new rest facilities were found to significantly reduce job-related stress [20].

ATCs with less than 40 h per week were 5.498 times (OR 5.498, 95% confidence interval [CI]: 1.451–20.840) more likely to receive a non-fit result (conditionally fit or unfit) compared to ATCs with over 52 h per week. This result implies that ATCs with health issues may be excluded from extended work over 40 h per week. According to Article 53 of the Labor Standards Act in Korea [21], employers are required to obtain employees’ consent for extended work, and workers possess the right to refuse extended work for health-related reasons. Furthermore, some companies impose restrictions on extended work for employees who need to recuperate from illness, injury, or fatigue [22]. In a comparable context, the working schedule was associated with the presence of sick leave, with two shifts showing a prevalence ratio of less than 1 when compared to fixed day shift (OR 0.155, 95% confidence interval [CI]: 0.025–0.973). These results also imply that ATCs with health issues may be excluded from participating in shift work. According to the shift work guidelines issued by the Ministry of Employment and Labor in Korea [23], employees with health issues identified in health examination results and assessment opinions are advised to work during the day rather than engage in shift work, without extended work.

In this study, gender was found to be statistically significant in relation to sick leave status based on the chi-square test but not in the binary logistic regression analysis. This discrepancy can be attributed to two main factors: Although the sample size of 220 air traffic controllers may be adequate for representing the target population, it could be insufficient for detecting statistically significant effects in a multivariable analysis such as binary logistic regression. In contrast, the chi-square test assesses the simple association between two categorical variables, making it less sensitive to sample size constraints. Therefore, the non-significant result in the logistic regression may be due to limited statistical power stemming from the sample size. Binary logistic regression accounts for multiple variables simultaneously, such as age, occupational characteristics, and health status, to assess the unique contribution of each variable. During this process, the independent effect of gender on sick leave status might have been diminished due to overlap or shared variance with other variables. For instance, variables like age or health conditions could have played a more significant explanatory role, reducing the relative influence of gender. This result suggests that while gender was significant when analyzed independently, its effect was weakened when other relevant variables were controlled for in the multivariable model.

Concerning aviation medical examination results, control service types exhibited a significant difference result (χ^2^ = 13.472, *p* = 0.001). The findings indicated that 81.4% of ATCs were classified as fit, while 18.6% received a non-fit result (conditionally fit or unfit). These figures are consistent with the 2023 data from the Korean Aerospace Medical Association [24], which reported that 83.6% of pilots were classified as fit and 16.4% as non-fit (conditionally fit or unfit). For ATCs, 80.2% were classified as fit and 19.8% as non-fit (conditionally fit or unfit) [24]. This level is also comparable to the 2022 general health examination results for general workers, where out of 10,039,345 individuals, 1,979,703 cases (19.7%) involved diagnosed illnesses (category D) [25]. For those deemed conditionally fit, regular treatment and health management are necessary to continue performing aviation duties. However, individuals classified as unfit are prohibited from engaging in aviation duties according to Article 42, Paragraph 1 of the Aviation Safety Act, which stipulates that air traffic controllers who do not meet the medical certification criteria must not participate in aviation operations [4]. Therefore, effective health management is crucial to avoid being classified as non-fit (conditionally fit or unfit) in the aviation medical examination.

In this study, no measurement variable was identified that could elucidate the causes of non-fit (conditionally fit or unfit) results in the aviation medical examinations of ATCs. According to the final report of the study on the introduction of a fatigue management system for air traffic controllers [26], the most frequently diagnosed health condition was sleep disorders among ATCs, accounting for 7.6%, followed by hypertension at 6.6%. Workers engaged in shifts or night shifts are perpetually exposed to conditions that disrupt their circadian rhythms, potentially leading to fatigue and sleep disturbances as a result of the disruption of their internal biological clock [27]. The majority of participants in this study were involved in shift work (89.1%) and reported working night shifts for six days or more (84.5%). According to a study by Ha et al., these work patterns induce changes in the homeostatic response of the autonomic nervous system, and in men, an increase in shift duration is significantly associated with elevated risks of hypertension, obesity, and dyslipidemia [28]. For women, irregular menstrual cycles have been reported during the first three months after starting shift work [29]. Additionally, this study found that the majority of ATCs (92.7%) work long hours, averaging over 40 h per week. The nature of the work, which involves sitting for extended periods while monitoring aircraft from a fixed location, may contribute to the risk of cardiovascular diseases [30]. According to the Aviation Medical Examiner’s Manual in Korea [6], uncontrolled hypertension, coronary artery disease, and arrhythmias such as atrial fibrillation are grounds for unfit results in aviation medical examinations. For those with existing chronic diseases, regular medical check-ups and consistent medication management are imperative [31].

In this study, the prevalence of sick leave was 16.4% in ATCs. Female participants exhibited a higher prevalence of sick leave (χ^2^ = 4.325, *p* = 0.038). According to a study by Ostby et al., women use sick leave more often than men and tend to take longer sick leave days [32]. Socially based hypotheses have suggested that women may be subject to higher levels of stress, possibly aggravated by the effect of the so-called “double-burden hypothesis”, the idea that working women also carry more of the burden at home [33]. Also, sickness absence in pregnancy has increased considerably in Norway over the last two decades, and it now accounts for about 25% of the gender difference in sickness absence [34].

Control service types showed a significant difference in sick leave (χ^2^ = 7.988, *p* = 0.018). This variable may have varying associations with the presence of sick leave. Approach controllers reported a greater frequency of sick leave. This position is tasked with maintaining safe aircraft operations during critical phases, experiencing considerable pressure [8,11]. Working region also showed a difference prevalence in sick leave. (χ^2^ = 10.410, *p* = 0.015). Due to the variations in sample size of ATCs across different regions, careful consideration is recommended in the interpretation of these results.

Concerning health management types, certain variables demonstrated a difference prevalence in sick leave, including smoking cessation (χ^2^ = 4.086, *p* = 0.043) and weight control (χ^2^ = 6.218, *p* = 0.013). Smoking and obesity are primary contributors to cardiovascular disease [28,35]. Therefore, health management with smoking cessation and weight control may affect the presence of sick leave.

The reason for hospital visit with fatigue or abnormal findings was reported in 18.9%, although this finding was not statistically significant in this study. In addition, respondents with insomnia in this study were unable to work for a maximum of 30 days.

According to interviews with different regions, ATCs in region D exhibited the highest demand for sleep management and expressed a need for professional counseling to address job-related stress. ATCs in region B demonstrated a strong interest in managing both fatigue and job stress [13]. There is a strong correlation between sleep disorders and burnout [36]. To improve sleep quality among shift workers, it is recommended to minimize night shifts, maintain a consistent shift schedule for 3–5 days to stabilize circadian rhythms, and rotate shift schedules in a clockwise direction [37]. The International Civil Aviation Organization (ICAO) Fatigue Risk Management System (FRMS), which has been extended to include air traffic controllers since 2020 in Korea, utilizes a data-driven approach aimed at continuously monitoring and managing safety risks associated with fatigue, grounded in scientific principles, empirical knowledge, and operational experience [38]. Lee’s study proposed an FRMS-based strategy for modifying night shift hours to address health issues arising from shift work. This strategy involves optimizing the allocation of night shifts by supplementing personnel as needed and diversifying shift structures, allowing workers to follow individualized schedules rather than adhering to a conventional two-shift (day/night) model [39].

A clear association for health management could not be established in this study. It seems that the wide confidence internals for some variables may be attributed to the small sample size rather than a true association.

Further studies utilizing larger sample sizes and objective data, such as aviation medical examination results and medical records of sick leave determined by a doctor, are required to enhance the validity and reliability of the findings.

This study, which utilizes secondary data for analysis, may have several limitations. The absence of necessary variables may result in an inability to measure the required concepts in the analysis. This study relied on self-reported data rather than objective measures, which means the possibility of recall bias cannot be excluded. The cross-sectional design limits the ability to establish temporal relationships between variables.

Despite these limitations, the significance of this study is found in its analysis of the health factors associated with the control service types, based on data from a nationwide sample of ATCs.

Based on the findings of this study, the following recommendations are proposed: Firstly, given the varying workload and characteristics of control service types, regular job rotation should be implemented to reduce monotony, improve job satisfaction, and provide opportunities for the development of diverse career experiences. Secondly, the Fatigue Risk Management System (FRMS) should be actively utilized to prevent human error and sleep disorders caused by fatigue among shift workers. Lastly, organizational support must be provided to enhance both the physical and mental health of air traffic controllers (ATCs).

## 5. Conclusions

This study confirms the association of control service types with aviation medical examination results and sick leave in air traffic controllers of Korea. Notably, approach controllers demonstrated a higher likelihood of receiving non-fit results and taking sick leave compared to their area controller counterparts. Recommendations for enhancing the well-being of air traffic controllers include implementing regular job rotation to alleviate monotony, actively utilizing the Fatigue Risk Management System (FRMS), and establishing organizational support measures to promote both physical and mental health.

## Figures and Tables

**Table 1:** Demographic, occupational, and health-related characteristics (N = 220).

Variables	Categories	N	%	Mean ± SD
Gender	Male	102	46.4	
	Female	118	53.6	
Age	20s	35	15.9	38.4 ± 8.8
	30s	90	40.9	
	40 and above	95	43.2	
Working region	Incheon	136	61.8	
	Daegu	52	23.6	
	Jeju	18	8.2	
	Others	14	6.4	
Control service types	Aerodrome control	73	33.2	
	Approach control	62	28.2	
	Area control	85	38.6	
Control service experience	<5	62	28.2	
(years)	5–10	64	29.1	
	>10	94	42.7	
Working hours	≤40	16	7.3	
(hours/weekly)	41–51	116	52.7	
	≥52	88	40.0	
Working schedule	2 shifts	128	58.2	
	3–4 shifts	68	30.9	
	Fixed day shift	24	10.9	
Number of night shifts	≤5	34	15.5	
(days/monthly)	≥6	186	84.5	
Hospital visit	Yes	106	48.2	
	No	114	51.8	
Reason for hospital visit	Treatment of existing diseases	54	50.9	
	Fatigue or abnormal findings	20	18.9	
	Abnormal physical symptoms	32	30.2	
Reason for hospital visit	Treatment of existing diseases	54	50.9	
	Fatigue or abnormal findings	20	18.9	
	Abnormal physical symptoms	32	30.2	
Health management	Yes	208	94.5	
	No	12	5.6	
Health management types	Smoking cessation	101	14.0	
(multiple answers)	Moderation in alcohol consumption	100	13.8	
	Physical exercise	149	20.6	
	Dietary improvement	78	10.8	
	Stress management	75	10.4	
	Weight control	90	12.4	
	Management of musculoskeletal diseases	9	1.2	
	Psychological counseling	4	0.6	
	Sleep management	54	7.5	
	Fatigue management	49	6.8	
	Other relevant practices	2	0.3	
	None	12	1.7	

**Table 2:** Characteristics of aviation medical examination results and sick leave (N = 220).

Variables	Categories	N	%	Mean ± SD
Aviation medical examination results	Fit	179	81.4	
	Non-fit	41	18.6	
Presence of sick leave	No	184	83.6	
	Yes	36	16.4	
Duration of sick leave				11.7 ± 13.7
Reasons for sick leave	Respiratory diseases	19	52.7	
	Gastrointestinal diseases	4	11.1	
	Orthopedic diseases	2	5.6	
	Otolaryngological diseases	2	5.6	
	Other conditions	9	25.0	

**Table 3:** Difference of aviation medical examination results by demographic, occupational, and health-related characteristics (N = 220).

Variables	Categories	Fit (n/%)	Non-Fit (n/%)	χ^2^	*p*
Gender	Male	87 (85.3)	15 (14.7)	1.937	0.164
	Female	92 (78.0)	26 (22.0)		
Age	20s	28 (80.0)	7 (20.0)	0.390	0.823
	30s	75 (83.3)	15 (16.7)		
	40 and above	76 (80.0)	19 (20.0)		
Working region	Incheon	109 (80.1)	27 (19.9)	2.707	0.439
	Daegu	44 (84.6)	8 (15.4)		
	Jeju	13 (72.2)	5 (27.8)		
	Others	13 (92.9)	1 (18.6)		
Control service types	Aerodrome control	65 (89.0)	8 (11.0)	13.472	0.001
	Approach control	41 (66.1)	21 (33.9)		
	Area control	73 (85.9)	12 (14.1)		
Control service experience	<5	50 (80.6)	12 (19.4)	0.301	0.860
(years)	5–10	51 (79.7)	13 (20.3)		
	>10	78 (83.0)	16 (17.0)		
Working hours (hours/weekly)	≤40	10 (62.5)	6 (37.5)	5.314	0.070
	41–51	93 (80.2)	23 (19.8)		
	≥52	76 (86.4)	12 (13.6)		
Working schedule	2 shifts	104 (81.3)	24 (18.7)		
	3–4 shifts	57 (83.8)	11 (16.2)	0.913	0.633
	Fixed day shift	18 (75.0)	6 (25.0)		
Number of night shifts (days/monthly)	≤5	28 (82.4)	6 (17.6)	0.026	0.872
	≥6	151 (81.2)	35 (18.8)		
Hospital visit	Yes	82 (77.4)	24 (22.6)	2.164	0.141
	No	97 (85.1)	17 (14.9)		
Reason for hospital visit	Treatment of existing diseases	39 (72.2)	15 (27.8)		
	Fatigue or abnormal findings	18 (90.0)	2 (10.0)	2.649	0.266
	Abnormal physical symptoms	25 (78.1)	7 (21.9)		
Health management ^1^	Yes	168 (91.7)	40 (8.3)		0.308
	No	11 (80.8)	1 (19.2)		
Health management types	Smoking cessation	86 (85.1)	15 (14.9)	1.764	0.184
(multiple answers)	Moderation in alcohol consumption	82 (82.0)	18 (18.0)	0.049	0.825
	Physical exercise	124 (83.2)	25 (16.8)	1.051	0.305
	Dietary improvement	68 (87.2)	10 (12.8)	2.696	0.101
	Stress management	63 (84.0)	12 (16.0)	0.522	0.470
	Weight control	77 (85.6)	13 (14.4)	1.765	0.184
	Management of musculoskeletal diseases	8 (88.9)	1 (11.1)	0.350	0.554
	Psychological counseling	4 (100.0)	0	0.933	0.334
	Sleep management	42 (77.8)	12 (22.2)	0.607	0.436
	Fatigue management	37 (75.5)	12 (24.5)	1.424	0.233
	Other relevant practices	1 (50.0)	1 (50.0)	1.309	0.253
	None	11 (91.7)	1 (8.3)	0.889	0.346

^1^ Fisher’s exact test.

**Table 4:** Differences in the presence of sick leave by demographic, occupational, and health-related characteristics (N = 220).

Variables	Categories	None n (%)	Sick Leave n (%)	χ^2^	*p*
Gender	Male	91 (89.2)	11 (10.8)	4.325	0.038
	Female	93 (78.8)	25 (21.2)		
Age	20s	27 (77.1)	8 (22.9)	1.583	0.453
	30s	75 (83.3)	15 (16.7)		
	40 and above	82 (86.3)	13 (13.7)		
Working region	Incheon	108 (79.4)	28 (20.6)	10.410	0.015
	Daegu	51 (98.1)	1 (1.9)		
	Jeju	14 (77.8)	4 (22.2)		
	Others	11 (78.6)	3 (21.4)		
Control service types	Aerodrome control	63 (86.3)	10 (13.7)	7.988	0.018
	Approach control	45 (72.6)	17 (27.4)		
	Area control	76 (89.4)	9 (10.6)		
Control service experience	<5	52 (83.9)	10 (16.1)	0.417	0.812
(years)	5–10	52 (81.2)	12 (18.8)		
	>10	80 (85.1)	14 (14.9)		
Working hours (hours/weekly)	≤40	15 (93.8)	1 (6.3)	2.691	0.260
	41–51	93 (80.2)	23 (19.8)		
	≥52	76 (86.4)	12 (13.6)		
Working schedule	2 shifts	110 (85.9)	18 (14.1)	1.885	0.390
	3–4 shifts	56 (82.4)	12 (17.6)		
	Fixed day shift	18 (75.0)	6 (25.0)		
Number of night shifts	≤5	29 (85.3)	5 (14.7)	0.081	0.776
(days/monthly)	≥6	155 (83.3)	31 (16.7)		
Hospital visit	Yes	75 (70.8)	31 (29.2)	24.802	0.000
	No	109 (95.6)	5 (4.4)		
Reason for hospital visit	Treatment of existing diseases	39 (72.2)	15 (27.8)	1.936	0.380
	Fatigue or abnormal findings	16 (80.0)	4 (20.0)		
	Abnormal physical symptoms	20 (62.5)	12 (37.5)		
Health management ^1^	Yes	175 (84.1)	33 (15.9)		0.310
	No	9 (75.0)	3 (25.0)		
Health management types	Smoking cessation	90 (89.1)	11 (10.9)	4.086	0.043
(multiple answers)	Moderation in alcohol consumption	83 (83.0)	17 (17.0)	0.054	0.816
	Physical exercise	125 (83.9)	24 (16.1)	0.022	0.882
	Dietary improvement	64 (82.1)	14 (17.9)	0.222	0.638
	Stress management	67 (89.3)	8 (10.7)	2.699	0.100
	Weight control	82 (91.1)	8 (8.9)	6.218	0.013
	Management of musculoskeletal diseases	8 (88.9)	1 (11.1)	0.189	0.664
	Psychological counseling	4 (100)	0	0.797	0.372
	Sleep management	43 (79.6)	11 (20.4)	0.839	0.360
	Fatigue management	39 (79.6)	10 (20.4)	0.753	0.385
	Other relevant practices	0	2 (100)	10.316	0.001
	None	9 (75.0)	3 (25.0)	0.692	0.406

^1^ Fisher’s exact test.

**Table 5:** Factors associated with the aviation medical examination results (fit vs. non-fit) of the population (N = 220).

Variables	Category	OR	95% CI	*p*
Gender	Male	0.708	(0.333–1.506)	0.370
(ref. female)				
Control service types	Aerodrome control	0.611	(0.218–1.715)	0.350
(ref. area control)	Approach control	3.044	(1.278–7.250)	0.012
Control service experience	<5	1.350	(0.532–3.425)	0.528
(ref. > 10 years)	5–10	1.347	(0.554–3.276)	0.511
Working hours	≤40	5.498	(1.451–20.840)	0.012
(ref. ≥ 52 h)	41–51	1.349	(0.605–3.008)	0.464
Working schedule	2 shifts	0.397	(0.076–2.085)	0.275
(ref. fixed day shift)	3–4 shifts	0.460	(0.083–2.556)	0.375
Number of night shifts	≥6	2.366	(0.472–11.844)	0.295
(ref. ≤ 5 days)				
Health management	No	1.879	(0.219–16.118)	0.565
(ref. yes)				

**Table 6:** Factors associated with the presence of sick leave among the population (N = 220).

Variables	Category	OR	95% CI	*p*
Gender	Male	0.532	(0.237–1.193)	0.126
(ref. female)				
Control service types	Aerodrome control	1.582	(0.557–4.487)	0.389
(ref. area control)	Approach control	3.891	(1.461–10.364)	0.007
Control work experience	<5	1.067	(0.394–2.886)	0.899
(ref. > 10 years)	5–10	1.310	(0.529–3.245)	0.559
Working hours	≤40	0.353	(0.038–3.293)	0.361
(ref. ≥ 52 h)	41–51	1.287	(0.575–2.881)	0.540
Working schedule	2 shifts	0.155	(0.025–0.973)	0.047
(ref. fixed day shift)	3–4 shifts	0.299	(0.045–1.972)	0.210
Number of night shifts	≥6	0.337	(0.051–2.208)	0.257
(ref. ≤ 5 days)				
Health management	No	2.365	(0.537–10.409)	0.255
(ref. yes)				

## Data Availability

The data generated during the study are available from the corresponding author on reasonable request.

## References

[1] Ministry of Land, Infrastructure and Transport in Korea (2023). 2023 Air Traffic Services Report.

[2] Han J.H., Shin H.Y., Jung S.J. (2022). 2022 Aviation Industrial Research and Analysis.

[3] Aviation Information Portal System Career Exploration. https://www.airportal.go.kr:450/airworks/menu.es?mid=a10403010000.

[4] Ministry of Land, Infrastructure and Transport in Korea Aviation Safety Act. https://www.law.go.kr/LSW/lsInfoP.do?lsiSeq=258789&ancYd=&ancNo=&efYd=20240717&nwJoYnInfo=Y&ancYnChk=0&efGubun=Y&vSct=%ED%95%AD%EA%B3%B5%EC%95%88%EC%A0%84%EB%B2%95#J42:0.

[5] Ministry of Land, Infrastructure and Transport in Korea Enforcement Regulations of the Aviation Safety Act. https://www.law.go.kr/LSW/lsInfoP.do?lsiSeq=264063&ancYd=&ancNo=&efYd=20240717&nwJoYnInfo=Y&ancYnChk=0&efGubun=Y&vSct=%ED%95%AD%EA%B3%B5%EC%95%88%EC%A0%84%EB%B2%95#J165240892024.

[6] Ministry of Land, Infrastructure and Transport in Korea (2024). Aviation Medical Examiner’s Manual.

[7] Caldwell J., Caldwell L., Mallis M., Miller J., Neri D., Paul M. (2009). Fatigue countermeasures in aviation. ASEM.

[8] Kim S.E., Baek K.W. (2017). Human-based aviation accidents with air traffic controller torts. KASLP.

[9] Orasanu J. Work schedules and fatigue management strategies in air traffic control (ATC). Proceedings of the Human Factors and Ergonomics Society Annual Meeting.

[10] Changa Y.H., Yangb H.H., Hsuc W.J. (2019). Effects of work shifts on fatigue levels of air traffic controllers. JATM.

[11] Chen Z., Liu W., Ding P., Wu Q. (2021). A field study of work type influence on air traffic controllers’ fatigue based on data-driven PERCLOS detection. Int. J. Environ. Res. Public Health.

[12] Ministry of Land, Infrastructure and Transport in Korea Regulations on Health Promotion Activities for Pilots and Air Traffic Controllers. https://www.law.go.kr/LSW/admRulLsInfoP.do?admRulSeq=2100000226070.

[13] Jung H.S., Jang J.S., Choi E.H., Baek E.M., Lee H.J., Choi Y.Y. (2024). Final Report on the Study on Establishing a Management System for the Physical and Mental Condition of Aviation Workers.

[14] Yun Y.J. (2017). The statistics about sick leaves among pilots in a civil airline (2016). KJAsEM.

[15] Allebeck P., Mastekaasa A. (2004). Chapter 5. Risk factors for sick leave—General studies. SJPH.

[16] Ministry of Land, Infrastructure and Transport in Korea Understanding of Air Traffic Service. https://www.molit.go.kr/atmo/USR/WPGE0201/m_37006/DTL.jsp.

[17] Ministry of Land, Infrastructure and Transport in Korea Air Traffic Service. https://www.molit.go.kr/USR/policyData/m_34681/dtl.jsp?search=&srch_dept_nm=&srch_dept_id=&srch_usr_nm=&srch_usr_titl=Y&srch_usr_ctnt=&search_regdate_s=&search_regdate_e=&psize=5&s_category=p_sec_7&p_category=&lcmspage=9&id=2502.

[18] Kim J.Y., Park N.S. (2021). Trends of Aircraft Safety Data and Analysis Methods. ETRI.

[19] Triyanti V., Azis H.A., Prasetyawan Y., Iridiastadi H., Yassierli (2020). Individual factors related to mental workload in air traffic controller. Convergence of Ergonomics and Design: Proceedings of ACED SEANES 2020.

[20] Kim Y.S., Park Y.S., Jo S.H. (2014). A Study on Occupational Stress of the VTS Operators. JNPR.

[21] Ministry of Government Legislation in Korea, Article 53 of the Labor Standards Act, Korean Law Information Center. https://www.law.go.kr/LSW/lsInfoP.do?lsiSeq=265959#AJAX.

[22] Korea Health Promotion Institute, Guidelines for Overtime Work. https://www.khepi.or.kr/kps.

[23] Ministry of Employment and Labor in Korea, Guidelines for the Application of Labor Standards Act for Shift Workers. https://www.moel.go.kr/policy/policydata/view.do?bbs_seq=5709.

[24] Aerospace Medical Association of Korea (2024). Aviation Medical Examination Results Statistics.

[25] Ministry of Employment and Labor in Korea (2023). Results of the 2022 Worker Health Examination.

[26] Kwon B.H., Han B.S., Ji Y.S. (2018). Final Report on the Study on the Introduction of a Fatigue Management System for Air Traffic Controllers.

[27] Harrington J.M. (2001). Health effects of shift work and extended hours of work. OEM.

[28] Ha M.N., Roh S.C., Park J.S. (2003). Shiftwork duration and metabolic risk factors of cardiovascular disease. Korean J. Occup. Environ. Med..

[29] Kim Y.H., Kim Y.M., Gu M.J., Kim S.H., Lee N.Y., Jang K.O. (2007). Experience of shifting workers. J. Korean Acad. Community Health Nurs..

[30] Franklin B.A., Rusia A., Haskin-Popp C., Tawney A. (2021). Chronic stress, exercise and cardiovascular disease: Placing the benefits and risks of physical activity into perspective. Int. J. Environ. Res. Public Health.

[31] Apgar T.L. (2016). Practical ways to improve medication adherence. Fam. Pract. Manag..

[32] Ostby K.A., Mykletun A., Nilsen A. (2018). Explaining the gender gap in sickness absence. Occup. Med..

[33] Nilsen W., Skipstein A., Ostby K.A., Mykletun A. (2017). Examination of the double burden hypothesis—A systematic review of work-family conflict and sickness absence. Eur. J. Public Health.

[34] Ariansen A.M., Mykletun A. (2014). Does postponement of first pregnancy increase gender differences in sickness absence? A register based analysis of Norwegian employees in 1993–2007. PLoS ONE.

[35] Korea Disease Control and Prevention Agency 9 Lifestyle Rules for Preventing and Managing Cardiovascular Disease. https://www.kdca.go.kr/contents.es?mid=a20303020300.

[36] Membrive-Jiménez M.J., Gómez-Urquiza J.L., Suleiman-Martos N., Velando-Soriano A., Ariza T., Fuente-Solana E.I., Fuente G.A. (2022). Relation between Burnout and Sleep Problems in Nurses: A Systematic Review with Meta-Analysis. Healthcare.

[37] Korean Academy of Sleep Medicine Shift Worker Sleep. https://www.sleep.or.kr.

[38] (2016). ICAO, Fatigue Management Guide for Air Traffic Service Providers, First Edition. https://www.unitingaviation.com/publications/FM-Guide-Air-Traffic-SP/?_gl=1*kybtup*ga*ODE5OTQ5MjkyLjE3MzEyNDQ2MjU.*_ga_992N3YDLBQ*MTczMTI0NDYyNC4xLjEuMTczMTI0NDc1Ny4wLjAuMA..#page=18.

[39] Lee Y.J. (2019). Domestic Application Plan of Fatigue Risk Management System by Air Traffic Controller. J. Adv. Navig. Technol..

